# Meeting the current and future challenges of oncology drug development

**DOI:** 10.3332/ecancer.2013.ed21

**Published:** 2013-05-23

**Authors:** Moira Howie, Kathy Oliver, Angela Timoney, Gordon McVie

**Affiliations:** 1 Eli Lilly and Company, Lilly UK, Surrey, UK; 2 International Brain Tumour Alliance, UK; 3 Scottish Medicines Consortium, UK; 4 European Institute of Oncology, Via G.Ripamonti, 435-20141 Milan, Italy

## Abstract

This breakout session highlighted four distinct perspectives from leading individuals within patient advocacy, industry, an appraisal committee and physicians on the future and challenges faced by targeted therapy in HTA evaluation. Bringing together leaders from key stakeholders in the process, it gave participants the opportunity to examine how the same HTA evaluation process is interpreted from multiple perspectives. The presentation of an industry supported “Six Nation Public Opinion Survey of Cancer Knowledge and Attitudes” provided detailed insight into how the general public, patients and caregivers view cancer alongside various available and possible future therapies. An interactive ‘perspectives activity’ session provided all participants with an opportunity to think through and discuss the HTA process, and its challenges, from the four distinct positions involved.

We declare that we have no conflicts of interest.

## Report on Day 2 Breakout Session 3

### The Current and Future Challenges of Targeted Therapy in HTA Evaluation

This breakout session gave participants four distinct perspectives on current and future challenges of targeted drugs and related diagnostics in HTA evaluation. Facilitated by Moira Howie (Eli Lilly, UK) and Chaired by Prof Gordon McVie (European Institute of Oncology, Italy) and involving Kathy Oliver (International Brain Tumor Alliance, UK) and Prof Angela Timoney (Scottish Medicines Consortium, UK), the breakout session highlighted the issue from the differing perspectives of industry, clinicians, a HTA appraisal committee as well as the viewpoint of a leading international patient advocacy group.

Prof Gordon McVie (European Institute of Oncology) presented the perspective of physicians relating to issues with HTA. It was stated that although randomised trials for medicines were deemed suitable, physicians felt devices were another matter. Physicians noted with concern the failure of biomarker research and the late application of imaging. Regarding targeted therapy, Prof McVie wondered whether, although patients were always at the ‘end of the agenda’, was targeted therapy really a ‘new deal’, while Prof Angela Timoney (Scottish Medicines Consortium) noted that targeted therapy may have particular assessment issues from the Appraisal Committee perspective.

Chair of the Scottish Medicines Consortium (SMC), Prof Angela Timoney, described the SMC as being a small organisation but made up of multiple stakeholders involving industry, patient and public representatives, and ‘budget holders’, highlighting that this variety both helped give the SMC its strength and also was a small step in the direction of ensuring the patient voice is heard by key stakeholders. It was noted that despite its size, at its core, activity for the SMC was the rapid health technology assessment of all new medicines in Scotland. The three main pillars for marketing approval by the EMA, and based mostly on the outcomes of clinical randomised phase III trials, were identified as Quality, Safety, and Efficacy. However before a medicine or treatment can be prescribed by HCPs and reimbursed in more countries, a fourth pillar of ‘relative effectiveness / cost effectiveness’ must be addressed. The SMC health technology assessments were described to the participants and included measuring the costs, benefits, and ‘disbenefits’ of new technologies. The attendees were told that the SMC favoured cost utility analysis (QALYs) and that although there is no QALY threshold, under £20,000 is usually an amount acceptable to the NHS and anything over £30,000 must be justified. Prof Timoney drew attention to the politics surrounding HTA, noting the discussions surrounding “promising new medicines” and the costs associated with small patient numbers and available ‘funds’ (i.e. Rarer Conditions Medicine Fund, Cancer Drugs Fund). On the challenges ahead for the appraisal body, Prof Timoney highlighted that although society (i.e. the payer) values severity, she questioned if it valued rarity. She noted that the increasing segmentation of the market does not seem to result in less funds per segment but interestingly, a paradoxical increase. It was posited that given increasing financial constraints, does society want this? It was felt that multiple perspectives were very necessary, and more discussion may be needed regarding the difference between ‘value’ and ‘values’.

Patient advocacy group representative Kathy Oliver (IBTA) demonstrated that although quantitative data, such as scientific, clinical and cost analysis, is the basis for some evidence, there should very much be a role for including qualitative information in HTA assessments; for example, the drug treatment’s effect on the patient’s quality of life and wellbeing. The patient perspective could place value and importance on widely different aspects in the process from the clinical perspective, for example, impact on their daily life and their own participation in wider society. This perspective could help shape a patient’s choice of a therapy and result in one being used with allows this preference to occur. For example, a chemotherapy taken by capsule at home may offer greater “value” to a patient because of the ease and convenience of administration. HTA should be amenable to reflecting the value of the patient perspective while still appreciating the overall standard focus on costs. It was also expressed that there could be the risk that targeted therapies become “exclusive medicine”, creating “haves” and “have nots” in the patient population, based on genetic profiling. To avoid this, efforts must also be made to conduct studies into other treatments for those whose genetic profiles are not suitable to a particular targeted therapy. The potential very high cost of targeted therapies will also be problematic for HTA.

As Director for Global Oncology Advocacy & Professional Relations and Global PACE with Eli Lilly, Moira Howie voiced the industry perspective and drew attention to the PACE (Patient Access to Cancer Care www.pacenetwork.com) initiative launched in 2011. Highlighting that Lilly’s vision was to ‘develop and acquire innovative medicines to improve outcomes for patients’ and that listening to the public and to patients themselves about personalised medicine often yields the best outcomes, the results of the 2012 “Six Nation, Public Opinion Survey of Cancer Knowledge and Attitudes” were summarised for the attendants. Over 4,300 individuals, including over 3,000 members of the general population, more than 600 cancer patients and more than 600 caregivers were surveyed from the United States, France, Germany, Italy, Japan and the United Kingdom. From the survey, the results showed that the public has a low awareness of personalised medicine. However once introduced to the concept, there was a noticeably higher level of willingness to undergo testing in support of personalised treatment, and a surprisingly high majority stated they would be willing to share their medical records and test results in pursuit of better treatments for themselves or to help others. The results showed an interesting range of perspectives on personalised medicine as the public believes that patients themselves will be the primary beneficiaries of personalised medicine, followed by physicians and then industry. Despite obvious variances in beliefs on who should pay the costs of treatments and personalised testing, majorities or near majorities in all countries believed that personalised medicine will end up saving health care systems money.

For Future Challenges in HTA evaluation from the industry perspective, Moira Howie (Eli Lilly) pointed out that the Global Survey findings established that the public and specifically cancer patients were lacking adequate information on personalised medicine. It was proposed that if patients and others had a higher awareness, this could fuel higher demand and increasing pressure on the decision makers and HTA to make medicines and new therapies available. As such, it could be seen as vital to ensure that developments of HTA would support timely access to personalised medicines. However, the limits of current HTA systems must also be understood, for example the lack of accepted standards on how to evaluate diagnostics and test treatment combinations. It was stated that HTA too needs to adapt to the new R&D paradigm and move away from ‘one off’ decision making towards a more dynamic and flexible approach. Regular and consistently high quality collaboration was highlighted as another suggested change in HTA moving forward: early and regular dialogue between manufacturers, EMA and HTA bodies; transparent education that is transferable between countries and individuals; and the participation of all stakeholders in defining the future of personalised medicine were considered as the heart of this collaboration.

An interactive ‘perspectives exercise’ (“Wearing someone else’s shoes”) concluded the breakout session, where participants were divided into 4 distinct groups representing patient advocacy, industry, HTA appraisal committee, and the clinical community. Each group discussed amongst themselves four different statements from their assigned stakeholders’ perspective for 5 minutes, before presenting their answers from their new assigned perspective. Having to place themselves in “someone else’s shoes” allowed for the sharing of a variety of responses from each of the groups and to challenge preconceived perceptions and thought processes associated with each stakeholder.

